# Supplementary material for: Transcriptome Analysis to Identify Genes Related to Flowering Reversion in Tomato
Source: Int J Mol Sci. 2022 Aug 12;23(16):8992. doi: 10.3390/ijms23168992 (PMC9409316; doi:10.3390/ijms23168992)
Supplement: Supplementary file 1 [file ijms-23-08992-s001.zip › Figure S3.pdf]

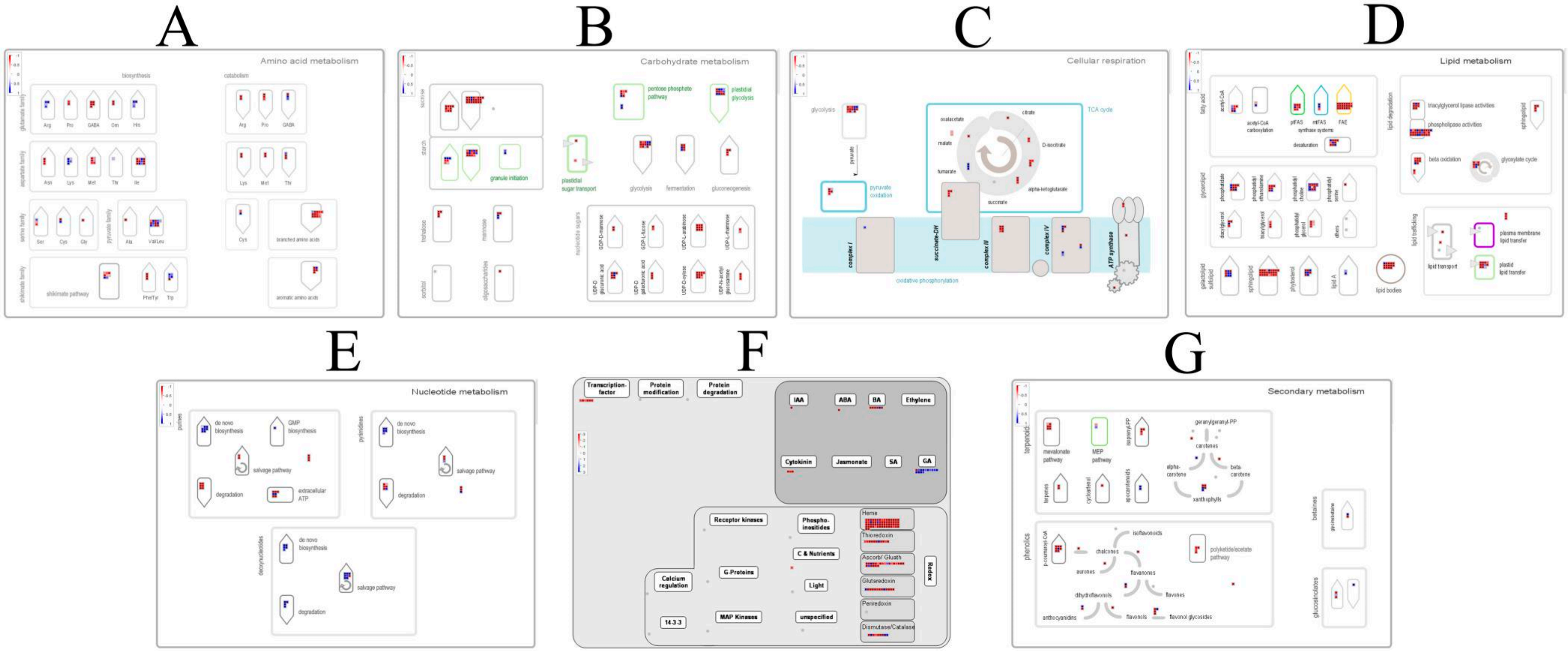

Figure S3. Enrichment analysis of DEGs generated by MapMan software. (A) Amino acid metabolism pathway. (B) Carbohydrate metabolism pathway. (C) Cellular respiration pathway. (D) Lipid metabolism. (E) Nucleotide metabolism. (F) Regulation overview. (G) Secondary metabolism.
