# Supplementary figures and images for: Transcriptome Analysis to Identify Genes Related to Flowering Reversion in Tomato
Source: Int J Mol Sci. 2022 Aug 12;23(16):8992. doi: 10.3390/ijms23168992 (PMC9409316; doi:10.3390/ijms23168992)

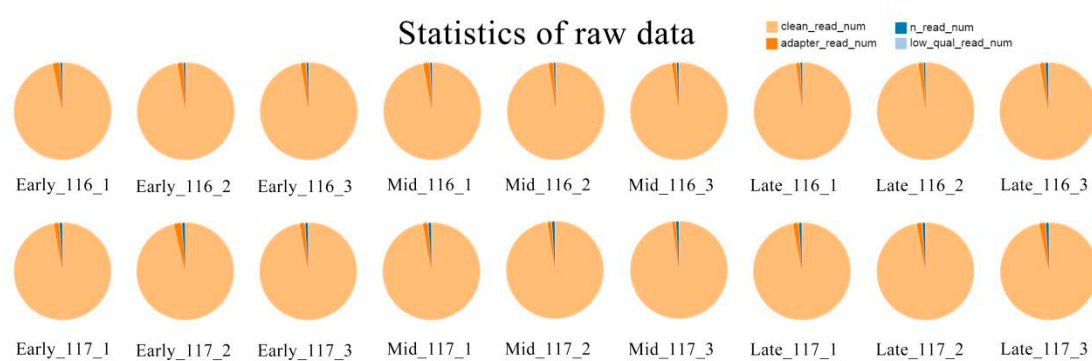

**Figure S1.** Statistics of raw data.

Supplement: Supplementary file 1 [file ijms-23-08992-s001.zip › Figure S1.pdf]

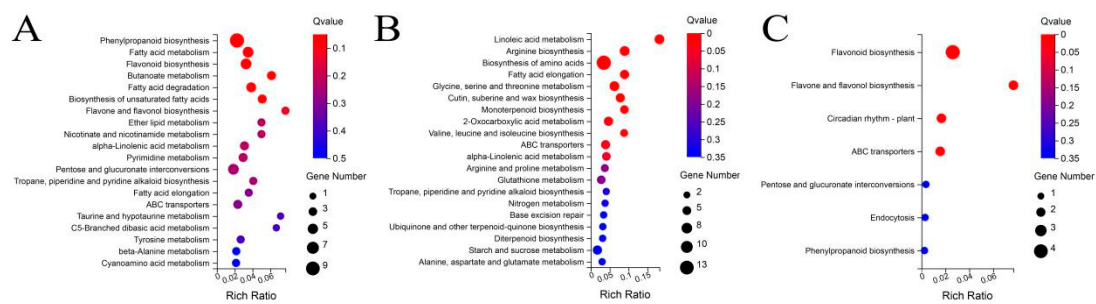

**Figure S2.** KEGG enrichment analysis in modules greenyellow (A), grey60 (B) and sienna3 (C) of WGCNA.

Supplement: Supplementary file 1 [file ijms-23-08992-s001.zip › Figure S2.pdf]
